# Evaluation of imaging features of pelvic echinococcosis based on multimodal images

**DOI:** 10.1186/s13018-020-01912-2

**Published:** 2020-10-26

**Authors:** Yu-Wei Chen, A. I. E. R. K. E. N. aikebaier, Yuan Zhao

**Affiliations:** Department of Imaging Center, First Affiliated Hospital of Xinjiang Medical University, 137 Liyushan Road, Urumqi, Xinjiang, 830054 China

**Keywords:** Pelvis, Echinococcosis, Computed tomography, Magnetic resonance imaging, X-ray computed, Management

## Abstract

**Background:**

Hydatid disease (HD) is a zoonotic parasitic disease caused by the larvae of Echinococcus, It is mainly prevalent in pastoral areas. Bone echinococcosis is rare, accounting for 0.5 to 4.0% of all echinococcosis. It is likely to miss the diagnosis and misdiagnose due to non-specific early symptoms and the clinical manifestations and imaging features. The clinical data of 15 patients with pelvic cystic echinococcosis were analyzed retrospectively, and the X-ray, CT, and MRI imaging features of the disease were discussed, which are reported below.

**Methods:**

All 15 patients underwent CT scan evaluation. A total of 8 patients underwent coronal, sagittal, and three-dimensional reconstruction with 3-mm-slice thickness, and 4 patients underwent X-ray plain film examination. Five cases underwent MRI scan. Eight cases underwent MRI or CT enhanced scan.

**Results:**

X-ray plain film is characterized by continuous cystic bone destruction, irregular low-density shadow when invading soft tissue, and sometimes calcification which can be seen on the wall or inside the cyst. The involved sacroiliac joint or hip joint may narrow or disappear. The involvement of pelvic cystic echinococcosis is relatively wide, and 80% of patients with pelvic cysts in this group had multiple lesions in the same period. Cystic expansive bone destruction was the most common. Pelvic CT revealed a lobulated hypodense lesion of varying size with internal septae, causing cortical thinning and destruction. Most of them had no periosteal reaction. The iliopsoas muscle is most easily invaded. Single cystic echinococcosis of pelvis showed intermediate or low signal intensity on T1-weighted images and hyperintensity on T2-weighted images in the involved bone and surrounding soft tissue on MRI, and the cyst wall showed linear low signal in T1WI, T2WI, and STIR sequences. The polycystic type is characterized by multiple cysts of varying signal intensity (daughter cysts) within a larger cyst is the typical MRI finding, forming “small vesicles” high signal daughter cysts. Osteosclerosis or calcification showed low signal in T1WI and T2WI.

**Conclusions:**

The results of this study suggest that the lesions of pelvic cystic echinococcosis are mostly cystic expansive and osteolytic bone destruction, which is easy to invade the surrounding soft tissue, often accompanied with calcification; among them, multiple cystic lesions are characteristic.

## Background

Hydatid disease (HD) is a zoonotic parasitic disease caused by the larvae of Echinococcus, also known as echinococcosis. It is mainly prevalent in pastoral areas, especially in Xinjiang, Qinghai, Ningxia, Gansu, Inner Mongolia, and Tibet. Bone echinococcosis is rare, accounting for 0.5 to 4.0% of all echinococcosis [1, 2]. It is likely to miss the diagnosis and misdiagnose due to non-specific early symptoms, and the clinical manifestations and imaging features are similar to those of bone tumor, bone cyst, and other diseases. The clinical data of 15 patients with pelvic cystic echinococcosis were analyzed retrospectively, and the X-ray, CT, and MRI imaging features of the disease were discussed, which are reported below.

## Materials and methods

### General information

Altogether, 15 patients with pelvic cystic echinococcosis were confirmed by histopathological examination or clinical and imaging examination in the first affiliated hospital of Xinjiang Medical University from February 2011 to June 2019, including 10 males and 5 females, aged 19–58 (37.7 ± 9.8) years. There were 4 cases of Kazak and 4 cases of Han nationality, 2 cases of Uygur, Hui and Mongolian each, and 1 case of Kirgiz. Fourteen cases had lived in Xinjiang for a long time, and one case had lived in Qinghai for a long time, with a course of 1–20 years. Clinical manifestations are the following: pain in the hip, lower limbs, and lumbosacral region in 5 cases and limitation of movement in 5 cases (including 1 case with nerve compression symptoms such as incontinence). Local infection occurred in 7 cases (1 case of subcutaneous mass of buttock, 4 cases of sinus formation after operation, 2 cases of non-healing incision). There were 1 case of chronic osteomyelitis, 3 cases of pathological fracture, and no symptoms in 2 cases. There were 7 cases of echinococcosis with other systems, 4 cases of pulmonary echinococcosis, 4 cases of hepatic echinococcosis, and 1 case of prostate echinococcosis. Among them, there were 7 cases confirmed according to the clinical history of echinococcosis combined with laboratory examination and imaging findings, and 8 cases were confirmed by histopathological examination.

### Methods

To record the imaging data of the patients according to the medical records, all 15 patients underwent CT scan evaluation (GE 16- or 64-slice CT machine). A total of 8 patients underwent coronal, sagittal, and three-dimensional reconstruction with 3-mm-slice thickness, and 4 patients underwent X-ray plain film (Philips DR system) examination. Five cases underwent MRI scan (Siemens 1.5T Avanto MR scanner). The scanning sequence included T1WI, T2WI, and short time inversion recovery (STIR); 8 cases underwent MRI or CT enhanced scan (MRI enhanced in 3 cases, CT enhanced in 5 cases), with iohexol (300 mg I/ml), 1.5 ml/kg, and injection flow rate of 3.0 ml/s; MRI enhanced contrast agent was GD DTPA, 0.2 ml/kg, injection flow rate 2.0 ml/s.

## Results

### Location and number of lesions

In 15 cases, sacrum, ilium, pubis, ischia, acetabulum, sacroiliac joint, hip joint, and soft tissue of surrounding muscles were the main affected parts. There were 4 cases of echinococcosis in the same side of femur and 3 cases of echinococcosis in the lumbar and sacral vertebra. The lesions occurred in 7 cases of the right pelvis, 6 cases of the left pelvis, and 2 cases of the bilateral pelvis. There were 38 lesions involving 3 or more parts in 7 cases, and 13 lesions invaded the surrounding soft tissue.

### Imaging findings

#### X-ray findings

**Fig. 1:**
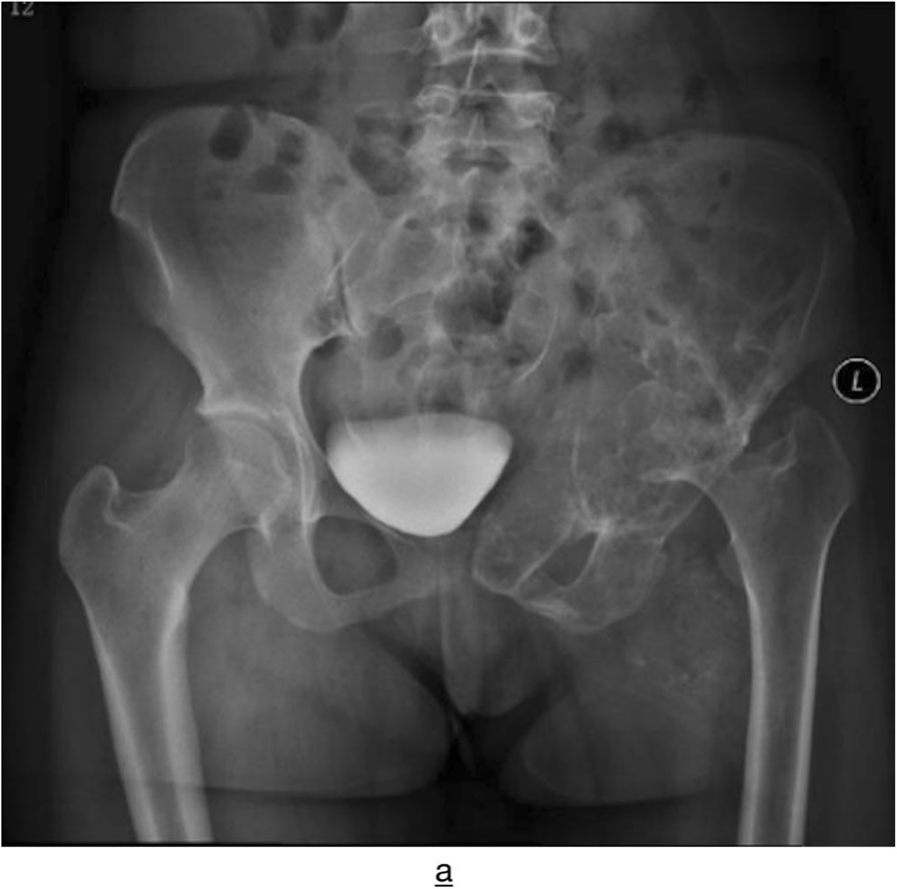
Radiograph showing osteolytic bone destruction of the left ilium, pubis, ischium, and femoral head, and subluxation of the left hip joint

All the 4 patients underwent X-ray examination showed multiple bone destruction, including 2 cases of single cystic expansive bone destruction, 1 case of irregular bone destruction with small bone shadow on the edge, and 1 case of cystic expansive bone destruction with patchy bone destruction; 4 cases of marginal osteosclerosis without periosteal reaction (Fig. 1). There were 4 cases of irregular low-density shadow in the surrounding soft tissue, 2 cases of irregular calcification shadow in the soft tissue, and sacroiliac joint narrowing in 3 cases, showing hyperostosis in the lower edge of sacroiliac joint and blurred articular surface.

#### CT findings

**Fig. 2:**
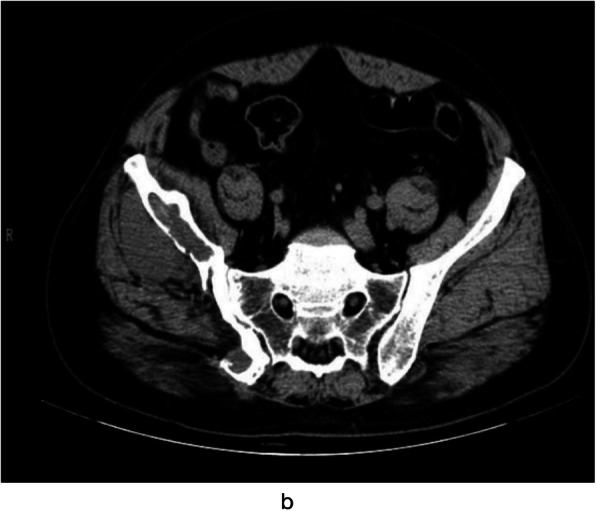
CT plain scan, showing cystic expansive bone destruction of the right iliac bone, right iliac bone and surrounding soft tissue, right pelvic wall and right gluteal muscles

**Fig. 3:**
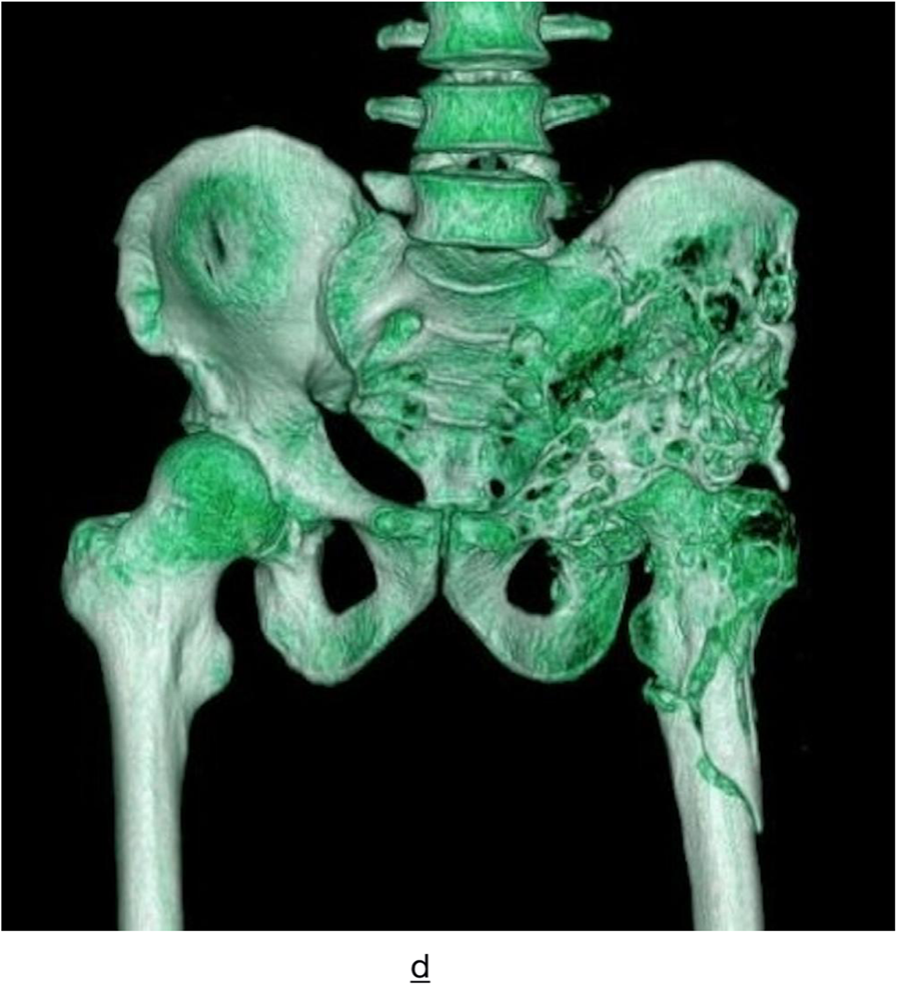
CT 3D reconstruction showing multiple wormlike bone destruction in the left margin of the sacrum, left ilium, pubis, and part of ischium, acetabulum, and upper femur, and the continuity of bone cortex of the left proximal femur was interrupted

**Fig. 4:**
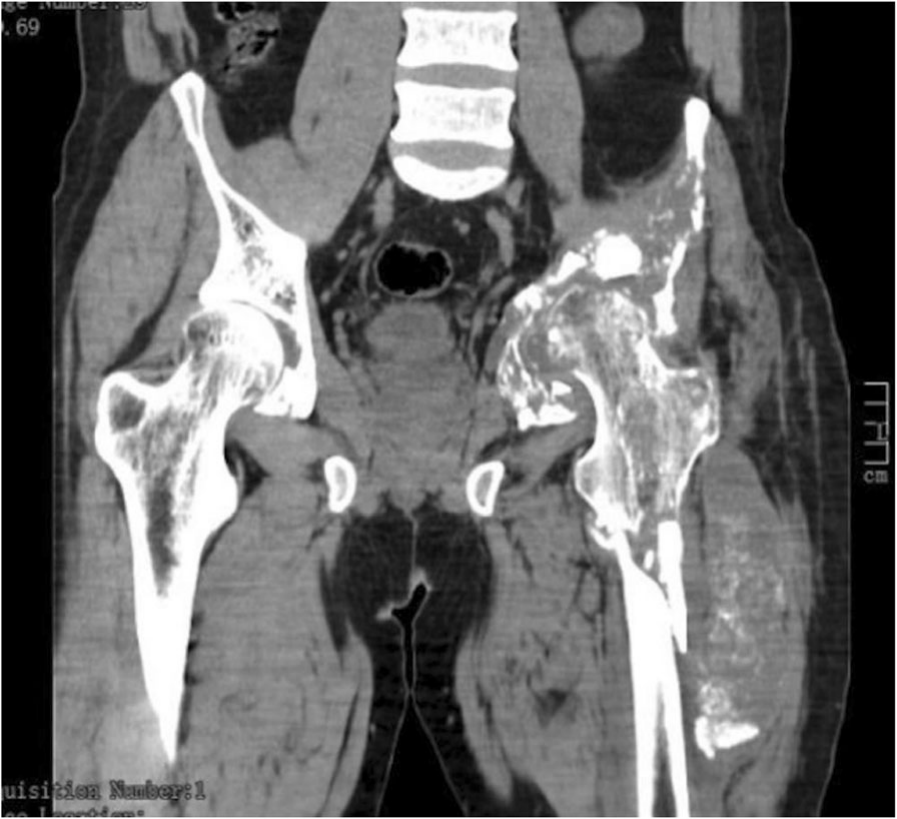
The CT coronal plane showed that the soft tissue around the upper left side of the femur was swollen, showing cystic changes, with irregular calcification in the cyst

CT scan was performed in all 15 cases, and different degrees of bone morphological changes after the removal of bone echinococcosis were found in 14 cases. Among them, there were 9 cases of cystic expansive bone destruction and thinning of bone cortex, and no periosteal reaction can be seen in the expansion area (Fig. 2), osteolytic bone destruction in 3 cases, erosive bone destruction in 3 cases (Figs. 3 and 4), and cystic expansive bone destruction and osteolytic bone destruction occurred in 1 case simultaneously. The lesions invaded the surrounding soft tissues (iliac muscle, iliopsoas, gluteal muscles, erector spinalis muscle, internal obturator muscle, etc.) in 13 cases. There were 10 cases of single cystic type and 4 cases of multicystic type. On plain CT scan, the boundary of the cyst showed round water-like, well-defined shadow, and the daughter cyst in the multicystic mother cyst showed septal round-like, low-density shadow, calcification inside the cyst or cyst wall in 9 cases, gravel-sand-like calcification in the cyst wall in 3 cases, shell-like calcification in 2 cases, irregular calcification in soft tissue in 4 cases. There were 7 cases of sacroiliac joint narrowing, 2 cases of sacroiliac joint disappearance, and 2 cases of multiple cystic lesions of sacroiliac joint.

#### MRI findings

**Fig. 5:**
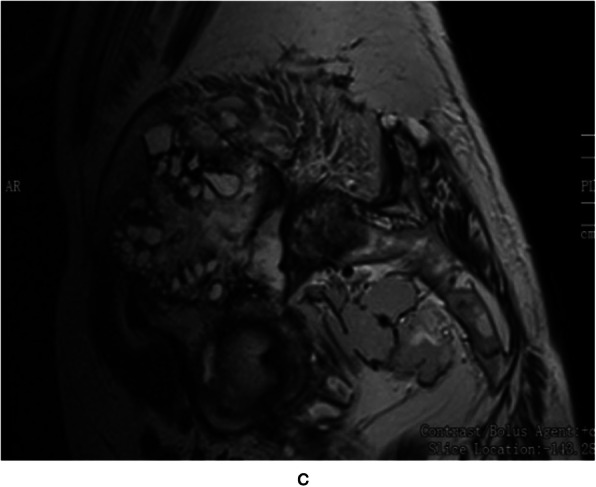
MRI sagittal T2 showed multiple rounded and irregular long T2 signals of different sizes

**Fig. 6:**
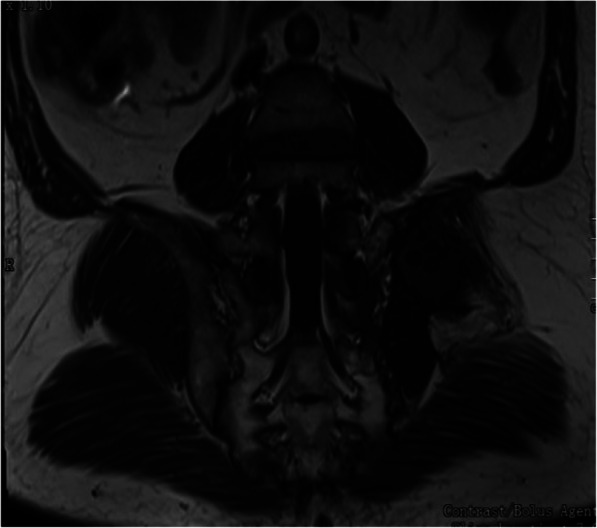
MRI coronal plane T1, which showing irregular low signal intensity of left ilium and iliopsoas muscle,and multiple circular lower signal intensity shadows can be seen in the low signal intensity shadow of the left iliac bone and iliopsoas muscle

A total of 5 patients underwent MRI examination. Among them, there were 2 cases of cystic expansive bone destruction, 2 cases of osteolytic bone destruction, and 1 case of erosive bone destruction. The lesions involved multiple parts. There were 3 patients of single cyst type, which showed intermediate or low signal intensity on T1-weighted images and hyperintensity on T2-weighted images, while in 2 cases of multiple cyst type, the mother capsule was filled with septate daughter cysts, and the signal intensity of daughter cysts was higher (Figs. 5 and 6). There was one case of arc calcification of cyst wall with hyperintensity on T1-weighted and T2-weighted. In one case of postoperative recurrent infection, MRI showed long T1 and mixed long T2 signal in the sacrum subcutaneous tissue, and the subcutaneous fat showed obvious heterogeneous enhancement.

#### CT or MRI enhancement features

In CT enhanced scan, there were no enhancement in 3 cases, a slight enhancement in 2 cases, and a linear enhancement of the cyst wall in 2 cases. In contrast-enhanced MRI scan, 3 cases showed slight linear enhancement at the edge of the lesion and partial septal enhancement and 1 case showed obvious heterogeneous enhancement of subcutaneous fat in the sacrum (indicating the presence of secondary infection).

## Discussion

### Clinical features and epidemic

*Echinococcus granulosus* is the main pathogen leading to hydatid disease in the pastoral areas of China, which seriously endangers human health and the development of animal husbandry. Granulosus has a carnivorous animal as a definitive host and an intermediate host such as sheep, cattle, and other herbivores. Humans are incidental hosts due to ingestion of the parasitic eggs from contaminated food or water. The larvae released from the eggs penetrate the intestinal wall and migrate to various organs. In addition to having a clear history of pastoral contact, a relatively higher prevalence has been noted in the rural populations that is mainly attributable to the slaughtering of animals in these areas [1]. In this study, we found that three patients were shepherds with a definite epidemiological history, and nine patients were all farmers. Pelvic bone disease is particularly difficult to diagnose, given the vague symptomatology and typically delayed clinical presentation. Hydatid lesions grow slowly; when located in the bone marrow cavity, they can spread along the cancellous bone to form bone destruction and cause pathological fracture. The lesions can penetrate through the bone cortex and invade the surrounding soft tissue to form a mass. When they break to the skin, chronic suppurative osteomyelitis can be developed by the formation of long-term unhealed fistulas and the outflow of hydatid detritus and pus. If the joint is involved, it can cause pathological dislocation. However, by the time clinical symptoms appear, such as pain, swelling, abnormal walking, incontinence due to nerve compression, paraplegia, and other related symptoms, the lesion is already large. Therefore, this disease warrants awareness and updated knowledge on part of clinicians, especially in endemic areas when patients present with generalized musculoskeletal complaints. Early radiologic investigations, laboratory evaluation, such as serological detection, and histopathologic analysis of the biopsy specimen are of paramount importance [3]. Serological tests are important in differential diagnosis. The immunoreaction of the human body is related to the hydatid cyst’s integrity, growth vigour, and location. Immunoreaction is heavier in ruptured hydatid cysts and lower when intact. Serological tests are frequently negative when the hydatid cyst is ageing, calcified, or dead. At present, the serologic examinations used in diagnosing hydatid cyst disease are classified into two categories. One is the detection of antigen from the hydatid fluid and protoscoleces, and the main antigenic components are Ag 5 and Ag B; the other is the detection of the antibody in the blood serum of patients. Specific antibody examinations are used for diagnosis including the following: the Casoni test, indirect hemagglutination (IHA), counterimmunoelectrophoresis (CIE), enzyme-linked immunosorbent assay (ELISA), and gold-labelled antibody [4]. Routine blood tests may reveal nonspecific inflammatory trends such as eosinophilia, deranged liver function tests, and raised CRP and erythrocyte sedimentation rate (ESR) [1]. In this group, only 5 cases underwent enzyme-linked immunosorbent assay (anti-EgcF antibody, anti-EgP antibody, anti-EgB antibody, and anti-Em2 antibody were all positive).

### Imaging features

X-ray plain film is one of the preferred methods for the examination of pelvic cystic echinococcosis, which is characterized by continuous cystic bone destruction, irregular low-density shadow when invading soft tissue, and sometimes calcification which can be seen on the wall or inside the cyst. The involved sacroiliac joint or hip joint may narrow or disappear. The radiological appearance of pelvic hydatidosis varies and is influenced mainly by the location of the cyst, age of the cyst, and associated complications, such as secondary infection and rupture, so definite diagnosis sometimes needs to be combined with clinical history and further imaging examination. Anterior-posterior pelvic radiograph showed that most of the 4 cases had multiple cystic expansive bone destruction and all of them had marginal osteosclerosis and no periosteal reaction. The surrounding soft tissue was invaded, and the soft tissue around the involved bone showed low-density spherical shadow of different size, with or without calcification. The sacroiliac joint involvement showed hyperostosis at the lower edge of the sacroiliac joint and blurred joint surface. Among them, 2 cases had a history of hepatic echinococcosis and a history of pulmonary echinococcosis. It is basically consistent with the relevant research results [4–6], which shows that X-ray examination is helpful in the diagnosis of pelvic cystic echinococcosis.

CT examination of pelvic cystic echinococcosis can not only precisely assess the type of bone destruction, calcification, and pathological fracture of echinococcosis [7], but also demonstrate the relationship with the surrounding tissue by three-dimensional reconstruction technique. Therefore, CT can be used as a common method for the diagnosis of pelvic cystic echinococcosis. The involvement of pelvic cystic echinococcosis is relatively wide, and 80% (12/15) of patients with pelvic cysts in this group had multiple lesions in the same period. Cystic expansive bone destruction was the most common (9/15). Pelvic CT revealed a lobulated hypodense lesion of varying size with internal septae, causing cortical thinning and destruction. Most of them had no periosteal reaction. Due to the thin pelvis cortex, the focus easily penetrates the cortex of the bone and invades the surrounding soft tissue, which mainly occurs in the gluteal muscle group, iliac muscle, iliopsoas, anterior iliac space, internal obturator muscle, and erector spinal muscle. According to the observation results of this group, the iliopsoas muscle is most easily invaded (7/15), and the soft tissue mass can spread along the iliopsoas muscle to form CT images similar to tuberculous abscess. When combined with femoral and spinal echinococcosis, the focus can invade the psoas major muscle or the muscle group around the upper part of the femur upward or downward. The presence of intralesional calcifications were found to be typical for hydatid bone disease and were useful in the differential diagnosis. As the focus of intraosseous echinococcosis can not form an external capsule, calcification often occurs in the surrounding soft tissue [7], showing gravel-like, shell-like, and irregular shape. In this group, 15 cases were examined by CT. The bone destruction showed thinning or osteolytic change of bone cortex in the expansion area and swelling of the surrounding involved soft tissue; rounded or oval cystic masses with sharp and thin edges and without contrast enhancement are typical. The cyst wall showed linear enhancement. There are various types of calcification, and all of them can occur inside or in the wall of the cyst. It is basically consistent with the relevant research results [4–6], which shows that CT examination is of great value for the diagnosis of pelvic cystic echinococcosis.

In addition, the recurrence rate of pelvic cystic echinococcosis is high, and the repeated course of disease can easily lead to secondary infection, or the interruption of treatment can lead to long-term wound healing, resulting in subcutaneous abscess or fistula [3], which requires long-term catheter drainage. Chronic osteomyelitis of the pelvis is rare, characterized by pathological changes such as dead bone, sinus, dead cavity, and cladding. CT can clearly show dead bone and intrabone abscess. Only 1 case was complicated with chronic osteomyelitis.

A total of 5 patients in this group underwent MRI examinations. As MRI examinations can observe the morphological characteristics, scope, location, and the relationship between the surrounding tissues and organs of bone cystic echinococcosis from the sagittal, coronal, and axial planes, it is of great value in the diagnosis of pelvic cystic echinococcosis. Single cystic echinococcosis of the pelvis showed intermediate or low signal intensity on T1-weighted images and hyperintensity on T2-weighted images in the involved bone and surrounding soft tissue on MRI [4] and high signal in fat suppression sequence, and the cyst wall showed linear low signal in T1WI, T2WI, and STIR sequences. The polycystic type is characterized by multiple cysts of varying signal intensity (daughter cysts) within a larger cyst is the typical MRI finding, forming “small vesicles” high signal daughter cysts [6]. Daughter cysts can be seen as cystic structures attached to the germinal layer that are hypointense relative to the intracystic fluid on T1-weighted images and hyperintense on T2-weighted images. After enhanced scanning, it could be marginal linear enhanced or septal partial enhanced, but there was no obvious enhancement inside the lesions. Osteosclerosis or calcification showed low signal in T1WI and T2WI. In addition, MRI has an important value for the ruptured infection of echinococcosis due to its good soft tissue resolution. When hydatid cysts are ruptured or infected, the signals of T1WI and T2WI are significantly enhanced due to the increase of protein content, especially T2WI, and the boundary of the cyst changed from sharp to blurred. It has been reported in the literature [5] that MRH (magnetic resonance hydrography) can clearly show the structure of the sinus when the lesion ruptures outside the skin to form a non-healing fistula and help to accurately assess the number of daughter cysts and degree of the disease.

### Management

In terms of management, hydatid disease of the pelvic bone is particularly a serious clinicopathologic entity as the cyst in this location may invade pelvic joints, which can potentially make complete recovery difficult. Although the definitive treatment of bony hydatidosis is surgery, a number of studies have highlighted the combination of antihelminthic chemotherapy and surgery as a feasible choice [2]. In the published medical literature, several surgical methods, including simple drainage or debridement, complete excision, total hip arthroplasty, bone grafting, and hemipelvectomy have been reported thus far [8]. Furthermore, bone cement filling is a reliable option to avoid the relapse of the cystic lesions due to its ability to kill the daughter cysts due to necrotizing effects of increased temperature in the polymerizing cement [9]. Among the collected cases, most of the patients were removed the lesion thoroughly and filled it with bone cement and took albendazole before and after operation. Hemipelvectomy was performed in patients with extensive pelvic bone invasion on one side. Fortunately, no recurrence has been observed since the operation in 2015. A patient who had a proximal fracture of the femur was performed bone cyst removal and tumor-type prosthesis replacement. Three patients received radiotherapy after surgery. Albendazole was used in the postoperative management of all patients. Chemotherapy using mebendazole or albendazole as only treatment is not adequate in most patients. However, it can be employed as neoadjuvant therapy to shrink the cyst load before surgery and/or as adjuvant therapy to decrease the recurrence risk [10]. However, the major goal of these procedures is to restore the limb function rather than complete eradication of the infectious etiology due to echinococcosis. In this study, most of them had a history of bone hydatid disease and had undergone at least one surgical treatment. Only 4 cases were secondary to liver hydatid or lung hydatid. Therefore, clinicians should perform a lifelong follow-up for early detection of potential recurrence and sequels.

### Differential diagnosis

Aneurysmal bone cyst, giant cell tumor, and bone tuberculosis can be considered as potential differential diagnoses in this case [3, 11]. The most common of these underlying lesions is the giant cell tumor. On conventional radiographs, giant cell tumors appear as eccentric epiphyseal lytic lesions with nonsclerotic but well-defined borders. MRI features are not specific, intermediate, or low signal intensity on T1-weighted images, and hyperintensity on T2-weighted images are common. Enhancement after intravenous gadolinium chelate administration may be seen. The aneurysmal bone cyst can be seen as an expanding lytic mass is seen on radiographs and CT images. It can appear a stepped liquid-liquid plane and diverticulum processes of different sizes. Bone tuberculosis generally invades the vertebral body and intervertebral disc at the same time, while bone echinococcosis often involves the paraspinal soft tissue on one side, and the intervertebral disc is rarely.

## Conclusion

The results of this study suggest that the lesions of pelvic cystic echinococcosis are mostly cystic expansive and osteolytic bone destruction, which is easy to invade the surrounding soft tissue, often accompanied with calcification. Among them, multiple cystic lesions are characteristic, which can improve the early diagnosis rate and achieve the purpose of early treatment.

## Data Availability

Not applicable.

## Abbreviations

MRI: Magnetic resonance imaging
CT: Computed tomography
MHR: Magnetic resonance hydrography
STIR: Short time inversion recovery
